# Isolation of Fungi and Bacteria Associated with the Guts of Tropical Wood-Feeding Coleoptera and Determination of Their Lignocellulolytic Activities

**DOI:** 10.1155/2015/285018

**Published:** 2015-08-26

**Authors:** Keilor Rojas-Jiménez, Myriam Hernández

**Affiliations:** ^1^Instituto Nacional de Biodiversidad, Apartado Postal 22-3100, Santo Domingo, Heredia, Costa Rica; ^2^Universidad Latina de Costa Rica, Campus San Pedro, Apartado Postal 10138-1000, San José, Costa Rica

## Abstract

The guts of beetle larvae constitute a complex system where relationships among fungi, bacteria, and the insect host occur. In this study, we collected larvae of five families of wood-feeding Coleoptera in tropical forests of Costa Rica, isolated fungi and bacteria from their intestinal tracts, and determined the presence of five different pathways for lignocellulolytic activity. The fungal isolates were assigned to three phyla, 16 orders, 24 families, and 40 genera;* Trichoderma* was the most abundant genus, detected in all insect families and at all sites. The bacterial isolates were assigned to five phyla, 13 orders, 22 families, and 35 genera;* Bacillus*,* Serratia,* and* Pseudomonas* were the dominant genera, present in all the Coleopteran families. Positive results for activities related to degradation of wood components were determined in 65% and 48% of the fungal and bacterial genera, respectively. Our results showed that both the fungal and bacterial populations were highly diverse in terms of number of species and their phylogenetic composition, although the structure of the microbial communities varied with insect host family and the surrounding environment. The recurrent identification of some lignocellulolytic-positive inhabitants suggests that particular microbial groups play important roles in providing nutritional needs for the Coleopteran host.

## 1. Introduction

Plant cell walls are predominantly composed of lignin, cellulose, and hemicellulose. Together, these three polymers represent one of the most abundant sources of renewable energy on Earth [1–3]. These polymers also constitute the basic nutrition source for a large number of terrestrial insects, of which the order Coleoptera is perhaps the most representative [4, 5]. The adaptation of the coleopteran insects to nutrient-limited diets, such as wood constituents, is attributed to the establishment of relationships with microorganisms. These microorganisms, highly prominent in the digestive tracts of the host, perform essential functions including digestion of lignocellulosic biomass, nutrient production, and compound detoxification [6–9].

Recently there has been an increasing interest in the gut microorganisms of wood-feeding Coleoptera, since this microbial-host interaction is highly relevant from several perspectives. For example, in natural ecosystems, beetles and their associated microorganisms perform important functions as prime contributors to the degradation of organic matter [10, 11]. Moreover, some species of beetles have become significant forest pests that cause large-scale economic losses. Therefore, a better understanding of their feeding capabilities is relevant for establishing novel management strategies [12–15]. From the biotechnological point of view, the coleopteran gut inhabitants represent a novel source for bioprospecting of enzymes related to the conversion of plant biomass into biofuels, production of industrial value-added products, and bioremediation of pollutants [16–18].

Most of the 300,000 beetle species described to date occur in tropical rainforests [19, 20]. Costa Rican rainforests, for example, are known to harbor approximately 10% of the species and 60% of the families of Coleoptera, including a number of wood-feeding beetles from the Scarabaeidae, Passalidae, Cerambycidae, Elateridae, and Tenebrionidae families [21]. The life cycles of these insects are highly seasonal, with most of the developmental stages occurring during the rainy season. The feeding sources of the adults include dung, carrion, and various plant parts, such as roots, stems, foliage, flowers, pollen, fruits, and seeds. Conversely, the diets of the larvae are more restricted to roots, decomposing organic matter, and decaying wood [11, 20–22].

The substrates on which the insects feed are major determinants for the gut microbial diversity. However, it is also possible that certain microbes have adapted to the endointestinal lifestyle and have developed mutualistic relationships necessary for the host survival. Hence, a fraction of the endosymbiont microbial community could be vertically transmitted [6, 7, 23]. Among the microbial groups, fungal and bacterial endosymbionts form complex communities that, besides the basic digestive functions, also perform nonconventional roles that include synthesis of vitamins and pheromones, nitrogen recycling, and resistance to pathogens, each of which has important implications for host fitness [8, 15, 24, 25]. In return, the insect provides a stable environment for the microbial growth with a steady intake of nutrients. Furthermore, the host can evolve to where tripartite beetle-fungi-bacteria mutualism takes place [23, 26]. This phenomenon seems to be widespread, although the mechanisms that govern these interactions are still poorly understood [4, 27].

Most of the previous studies on the microbial diversity of the coleopteran gut highlighted either bacterial diversity or, to a lesser extent, fungal diversity, generally obtained from a small number of economically important beetle species. In addition, some studies used metagenomic profiling for the discovery of novel lignocellulolytic genes. The purpose of the present work was to isolate and describe the composition of the cultivable fungi and bacteria associated with the guts of wood-feeding larvae of five families of Coleoptera and to determine their lignocellulolytic activities. For this, we collected larvae in tropical wet forests of several national parks in Costa Rica, isolated both fungi and bacteria from their guts, performed bioinformatics analysis, and assayed for the presence of five enzymatic activities related to the degradation of lignocellulosic materials. This work represents an initial step toward understanding relationships existing among the fungi, the bacteria, and the beetle host and the surrounding environment in a country having a particularly large biodiversity of Coleoptera.

## 2. Material and Methods

### 2.1. Insect Sampling

This study was conducted in tropical wet forests of 10 protected areas of Costa Rica with the respective permit resolutions R-CM-INBio-40-2008-OT and R-CM-INBio-48-2008-OT of the National Authority of the Ministry of Environment. At each site, approximately 3 km of natural trails was explored, looking for decaying fallen trees within 25 m on each side of the path. The sampling area represented nearly 150,000 m^2^ (or 0.15 km^2^) per national park. Every fallen tree found was exhaustively examined for the presence of galleries of wood-feeding beetle larvae, particularly from the Scarabaeidae, Passalidae, Elateridae, Cerambycidae, and Tenebrionidae families. The selected national parks covered most of the natural distribution of the five coleopteran families studied: wet tropical forest ranging from zero to 1,300 m altitude (Table 1).

The observed frequency of fallen trees and the presence of insect galleries within them varied according to the conditions of each site. Therefore, sample collection and study results were normalized to unit area. Most of the insect galleries contained insects of only one coleopteran family, but in few cases, two or three different families were present. The insect larvae, in the late second or third larval instars, were collected, placed in polyethylene boxes together with pieces of their feeding wood, and kept at ambient temperature until being transported to the laboratory (Table 2). The initial identification of the larval families was performed on site by a trained collector and later confirmed by the coleopteran taxonomist of the National Institute of Biodiversity. Once in the laboratory, the larval specimens were chilled at −20°C for 10 minutes, surface sterilized with ethanol, and then dissected in a sterile laminar flow hood.

### 2.2. Isolation of Bacteria and Fungi

The entire gut was removed from each larva and placed on a sterile Petri dish, crushed, and spread onto three different media plates. For isolation of fungi we used Potato-Dextrose Agar (PDA, Difco) amended with chlortetracycline (120 mg/L) and streptomycin (120 mg/L). For the isolation of bacteria we used one-third strength Luria-Bertani medium (3 g/L peptone, 5 g/L yeast-extract, 10 g/L NaCl, and 15 g/liter agar, pH 7.0) and the self-developed medium called LIGNO (1.5 g/L KH_2_PO_4_, 1.75 g/L K_2_HPO_4_, 0.8 g/L KNO_3_, 0.5 g/L MgSO_4_, 1 mL/L CaCl_2_ 0.1 M, 4 g/L sawdust, 2 g/L bagasse powder, and 17 g/L agar, pH 7.0). Plates were incubated at 28°C for up to three weeks and checked every other day regularly for visible microorganism growths. Each emerging fungus was transferred into a fresh PDA plate amended with the antibiotics mentioned above, while each emerging bacterial colony was replated into Luria-Bertani medium (Difco). An initial screening, based on the morphological traits of the fungi, was performed to discard redundant isolates from the same sample (characteristics such as the color, shape, border type, mycelial density, presence-absence of secretions, and growth rate were compared). A second screening was based on molecular taxonomy. The resulting nonredundant isolates were included in a database with associated information and were preserved in the National Biodiversity Institute's culture collection.

### 2.3. Lignocellulolytic Assays

We screened all the isolates for the presence of five different pathways for lignocellulolytic activity possibly associated with degradation of structural wood components, including cellulose, lignin, *β*-D-xylan, *β*-D-cellobiose, and *β*-D-glucans. These assays were performed by addition of specific substrates to the medium, or directly on the microbial culture, and hydrolysis was determined by a color change. All of the microorganisms were assessed at least two times for each enzymatic screening. The degradation of cellulose was determined using carboxymethylcellulose (CMC, Sigma) as the sole carbon source in the medium followed by staining with Congo red. Briefly, the bacterial isolates were grown for 48 h and fungi for 72 h, at 28°C on CMC medium (0.94 g/L KH_2_PO_4_, 1.9 g/L K_2_HPO_4_, 1.6 g/L KCl, 1.43 g/L NaCl, 0.15 g/L NH_4_Cl, 0.037 g/L MgSO_4_-7H_2_O, 0.017 g/L CaCl_2_, 0.1 g/L yeast-extract, 7.5 g/L CMC, and 15 g/L agar, pH 7.0). After this incubation period, the microorganism-containing agar plates were flooded with 0.05% Congo red for 10 min, until the dye bound CMC. The reaction was fixed with NaCl (50 mM) for 5 min and then rinsed with distilled water. The zones where the microorganism hydrolyzed the CMC were visible as clear halos [28]. The oxidative degradation of lignin was determined based on the decolorization of the dye Remazol Brilliant Blue R (RBBR, Sigma) when grown on solid media [29–31]. Plates with MEA-RBBR medium (20 g/L malt extract, 15 g/L agar, and 0.02% wt/vol RBBR, pH 7.0) were inoculated with the bacterial and fungal isolates and incubated at 28°C. At daily intervals for a period of 14 days, the plates were checked for the presence of a decolorized area around the colony or mycelia. Determination of the *β*-glucosidase, *β*-xylanase, and cellobiose hydrolase activities was performed using as substrates 10 mM 4-nitrophenyl *β*-D-glucopyranoside (Sigma), 4-nitrophenyl *β*-D-xylopyranoside (Sigma), or 4-nitrophenyl *β*-D-cellobioside (Sigma) dissolved in 50 mM ammonium acetate buffer, pH 5.0, amended with 0.7% of agar, and kept at 55°C. A drop of these solutions was placed directly on the bacterial colonies or fungal mycelia followed by incubation for 30 min at room temperature. The catalytic action of the microbial enzymes on the substrate was detected by the development of a yellow coloration, produced by the release of the* p*-nitrophenol group [30, 32].

### 2.4. Molecular Analyses

All the isolates were grown in Petri dishes containing the same media used for preservation. For the fungal DNA extraction, 400 mg of mycelia was ground with mortar and pestle in liquid nitrogen and further extracted using the DNeasy Plant kit (Qiagen, USA), including a pretreatment step consisting of the incubation at 60°C for one hour with 400 *μ*L of lysis buffer and 30 *μ*L of Proteinase K (20 mg/mL, Sigma Aldrich, USA). The bacterial DNA was extracted following the instructions of the NucleoSpin Tissue DNA Extraction kit (Macherey-Nagel, Germany). The fungal ITS1-5.8S-ITS2 regions were amplified by PCR from the total DNA using as forward primer the ITS1 5′-TCCGTAGGTGAACCTGCGG-3′ and the reverse primer ITS4 5′-TCCTCCGCTTATTGATATGC-3′ [33] with the following reaction program: 95°C for 10 min, 35 cycles at 94°C for 1 min, 54°C for 1 min, 72°C for 1 min, and additional extension at 72°C for 10 min. The 16S rRNA gene was amplified using primers 27f and 1492r [34] with the following program: 95°C for 10 min and 35 cycles of 94°C for 1 min, 52°C for 1 min and 72°C for 1 min, and 10 min extension at 72°C. The PCR products were purified using the NucleoSpin Extract II kit (Macherey-Nagel, Germany) according to manufacturer's protocol. Sanger sequencing of the samples was performed at the sequencing facility of the Dana Farber Cancer Institute at the Harvard University, Boston, Massachusetts, using the abovementioned forward and reverse primers for fungi and primers 27f and 785r for bacteria. Sequences were assembled using Seqman program of DNASTAR Lasergene 8.0 (GenBank accession: GU827479-GU827553, HM770962-HM771112).

### 2.5. Taxonomy

The taxonomic assignment of the bacterial sequences was performed by comparing the database against the 16S rRNA reference set 10 implemented in the Classifier tool of the Ribosomal Database Project, which assigned the 16S rRNA sequences to corresponding taxonomical hierarchy based on a naïve Bayesian rRNA classifier [35]. The taxonomy of the fungi was inferred by comparing the ITS1-5.8S-ITS2 sequences against the Warcup Fungal ITS trainset 1, a curated reference dataset implemented in the Classifier tool of the Ribosomal Database Project [35]. Every fungal taxonomical assignment was verified against the Index Fungorum (http://www.indexfungorum.org/) and appropriately corrected when synonyms or current names were identified.

### 2.6. Ecological Analyses

The analysis of the microbial communities was performed using the Vegan package implemented in the statistical programming environment and language R [36]. For this, a table with the taxonomic classifications to the levels of order, class, subphylum, phylum, and subkingdom of the fungal isolates was converted to taxonomic pairwise distances with the function* taxa2dist* and using variable step lengths between successive categories, proportional to the number of groups within each taxonomical level. This distance matrix was then used to construct a hierarchical clustering tree with the function* hclust* and the UPGMA distance method. A second matrix with the abundance distribution of the fungal isolates per insect family was prepared with the larval families in the rows, the fungal orders in the columns, and cells containing the counts of isolates in each taxon. This matrix was used to calculate Bray-Curtis distances between the insect fungal communities with the function* vegdist*. The advantage of this approach is that Bray-Curtis measures avoid the double zero problem: accounting for absences that are not indicators of similarities between sample units [37]. The generated distance matrix was used to cluster similarities between the microbial compositions of the larval families with the* hclust* function. The function* tabasco* was used to display compact integrated community information, plotting the fungal taxonomical relationships in the rows, the similarities between the insects' microbial compositions in the columns, and a heatmap with the respective abundance distribution [36]. The function* cca* was used to perform canonical correspondence analysis of the communities associated with the coleopteran families. A similar procedure was performed to analyze the community composition of bacteria and also for detecting differences among national parks.

## 3. Results

### 3.1. Taxonomic Composition of the Fungi and Bacteria Isolated

In this study, we isolated 92 fungal strains and 135 bacterial strains from larvae of five families of Coleoptera that were feeding on decaying wood in tropical wet forests of 10 national parks of Costa Rica. The 92 fungal isolates were assigned to three phyla, 16 orders, 24 families, and 40 genera (one different genus every 2.3 isolates). Within the phylum Zygomycota, we isolated members of the order Mucorales and within the phylum Basidiomycota members of the orders Agaricales, Polyporales, and Trichosporonales. Most of the fungi isolated from the gastrointestinal tracts of the larvae belonged to the phylum Ascomycota (89% of total). They were distributed in 12 orders, with Hypocreales being the dominant one; it comprised nearly 55% of the isolates (Table 3). The genus* Trichoderma* was the most abundant; it was the only one associated with all five families of Coleoptera and also present in each national park sampled.

Most fungal orders and genera were sparsely represented, with 68% of the orders and 55% of the genera found associated with a specific coleopteran family at a particular site. All the insect families also presented unique fungal isolates per site, with Tenebrionidae being the only coleopteran family in which all isolates were phylogenetically distinct. Regarding the sampling sites, Tenorio National Park showed the highest number of unique phylotypes, but Piedras Blancas National Park contained a more phylogenetically diverse array of isolates.

The 135 bacterial isolates were classified within five phyla, 13 orders, 22 families, and 35 genera (one different genus from every 3.8 isolates), including members of Actinobacteria, Proteobacteria, Firmicutes, Flavobacteria, and Fusobacteria. Approximately 82% of the bacteria belonged to *γ*-Proteobacteria and Firmicutes, accounting for 44% and 38% of the isolates, respectively. Within the *γ*-Proteobacteria, the genera* Serratia* and* Pseudomonas* were abundant, being present in all host families studied. Within Firmicutes, the genus* Bacillus* was clearly the most dominant. This single genus, which accounted for 20% of all the isolates, was a common gut inhabitant of all the insect families and was present at almost all the sites sampled (Table 4).

The remaining bacterial classes obtained in this study were less represented. For example, members of Actinobacteria accounted for 11% of the isolates whereas *α*- and *β*-Proteobacteria, Flavobacteria, and Fusobacteria represented less than 6% of the isolates. When calculating the percentage of isolates that were specific to a single site and host family, results showed that 42% of the isolates exhibited this characteristic, while the remaining 58% of the genera presented a broader host-site range. A small number of the genera were found in one site but in different host families (i.e.,* Rhizobium* in Hitoy Cerere National Park); others were associated with the same beetle family but in different sites (i.e.,* Leucobacter* with Passalidae). Results of the analysis of unique phylotypes per site and insect family showed that Hitoy Cerere National Park and Elateridae, respectively, presented the highest percentages of single bacterial isolates.

### 3.2. Lignocellulolytic Activity Determination

Nearly 65% of the fungal genera and 48% of the bacterial genera presented positive results in at least one of the five lignocellulolytic activities evaluated, with carboxymethylcellulose degradation being the most common activity observed in both groups (Table 5). In general, fungi showed more capability for degrading lignocellulosic materials than bacteria, with genera such as* Trichoderma*,* Bionectria,* and* Trametes* showing positive results in all the assays performed. Within bacteria,* Bacillus*,* Enterobacter,* and* Acinetobacter*, some of the most abundant genera isolated from the larval guts, tested positive for four out of the five enzymatic activities assayed: cellulase, *β*-glucosidase, *β*-xylanase, and cellobiose hydrolase activities. However, neither these genera nor any other bacterial group screened were able to degrade the Remazol Brilliant Blue molecules, while 30% of the fungal genera tested positive for this lignin-related degradation activity.

### 3.3. Comparison of Gut Inhabitants between Families of Coleoptera

**Figure 1:**
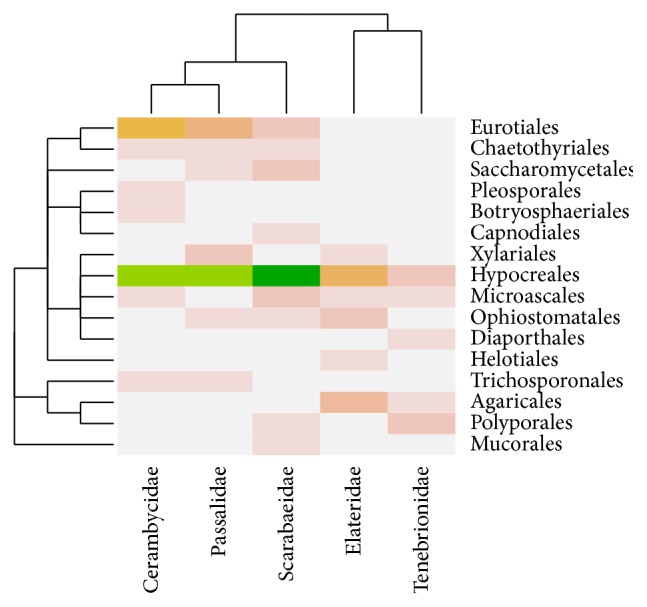
Heatmap of the abundance distribution of fungal communities associated with the guts of five wood-feeding families of Coleoptera. The taxonomic relationship of the fungal genera is shown in the rows, while the clustering of the coleopteran families, determined by their composition similarities, is shown in the columns. Higher intensities of the color reveal higher abundances of the isolates.

**Figure 2:**
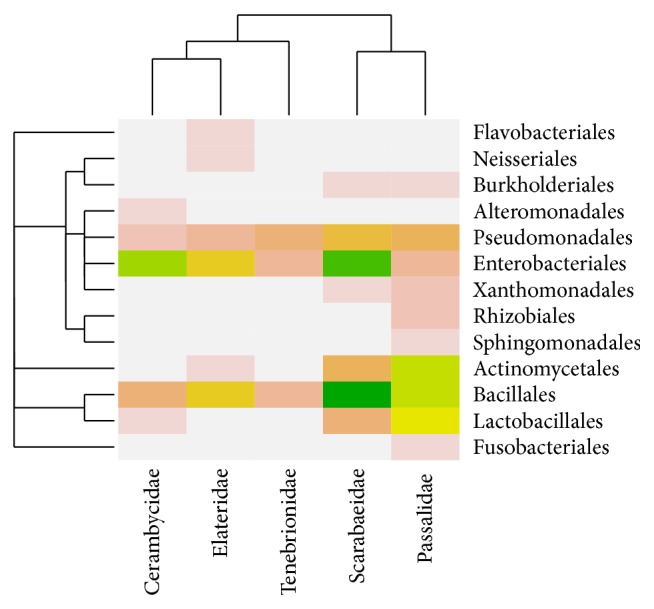
Heatmap of the abundance distribution of bacterial communities associated with the guts of five wood-feeding families of Coleoptera. The taxonomic relationship of the bacterial genera is shown in the rows, while the clustering of the coleopteran families, determined by their composition similarities, is shown in the columns. Higher intensities of the color reveal higher abundances of the isolates.

We performed community analysis with Vegan to gain insight into how the microbial gut composition of the beetle families related to one another. This approach clustered the environments according to Bray-Curtis distances of the abundance distribution of the isolates, considering also their phylogenetic relationships. The results showed that the fungal composition of the isolates associated with larvae of Cerambycidae, Scarabaeidae, and Passalidae clustered together; Cerambycidae and Passalidae shared one order of Basidiomycota and three orders of Ascomycota, while Scarabaeidae and Passalidae had in common four orders of Ascomycota. A second cluster was formed by the fungal microbiotas isolated from Tenebrionidae and Elateridae; they shared two orders of Ascomycota and one of Basidiomycota (Figure 1). The analysis of the bacterial dataset showed that the microbial compositions associated with Scarabaeidae and Passalidae formed part of the same cluster, sharing isolates belonging to *β*- and *γ*-Proteobacteria, Actinobacteria, and Firmicutes. The second cluster was formed by Tenebrionidae, Elateridae, and Cerambycidae that shared isolates assigned to Pseudomonadales, Enterobacteriales, and Bacillales (Figure 2). In addition, we performed canonical correspondence analysis for exploring relationships between the microbial communities of the coleopteran hosts. Results of this analysis where consistent with results obtained with the Bray-Curtis clustering for both the fungal and bacterial communities (Figure S1 in Supplementary Material available online at http://dx.doi.org/10.1155/2015/285018).

## 4. Discussion

We collected larvae of five families of wood-feeding Coleoptera in tropical forests of Costa Rica with the aim of estimating the species composition of cultivable fungi and bacteria inhabiting their guts and to identify microorganisms with relevant lignocellulolytic activities. The main limitation of this study is that the cultivation-dependent approach, based on artificial media, covers only a small proportion of the total microbial diversity present in this particular niche. The positive trade-off of this approach was the identification of several isolates with lignocellulose-degrading capabilities, which can be further used for the respective enzyme characterization, for direct degradation assays on residues from agriculture and forestry, for the treatment of industrial effluents, and for bioprospecting novel metabolites with other biotechnological applications. Despite the inherent bias of the isolation method, our results suggest that gut microbiota of wood-feeding tropical beetles presents a relatively high diversity in terms of microbial richness, phylogenetic composition, and lignocellulolytic activities.

The order Hypocreales represented about 60% of the total number of fungal isolates. Within this group, the genus* Trichoderma* was the most abundant, comprising nearly a quarter of the fungal collection. This genus was a common gut inhabitant of beetle larvae regardless of the host family or the geographic location. The reason for this dominance is not entirely clear; however, one possible explanation is that several species belonging to this fungal genus contain a number of glycoside hydrolases, peroxidases, laccases, and phenol oxidases, among other enzymes related to the degradation of lignocellulose materials. This feature might provide some advantages for using the recalcitrant polymeric materials passing through the gastrointestinal tract [16, 38–40].

In addition, our data indicate that guts of wood-feeding larvae were from environments having a high representation of Hypocreales, as also observed in a similar study performed in other locations of Costa Rica [41]. This is relevant for bioprospecting purposes, since wood-feeding beetles might constitute a good source of* Trichoderma*,* Metarhizium*,* Metacordyceps*,* Bionectria*, and other fungal genera known to possess a wide array of biotechnological applications [42–45]. The remaining orders presented a lower abundance and, in most of the cases, were represented by a single genus. Nevertheless, many of the genera showed the ability to degrade lignocellulose-related hexoses and pentoses, as also shown in other studies [46–50]. Within the phylum Basidiomycota, the genus* Trametes* showed positive results in all the lignocellulolytic assays related to the degradation of structural wood components. This white-rot fungus is a known model for studying degradation of lignin in free-living conditions and in this work reported in its association with the gut microbiota of wood-feeding insects [51, 52].

It is difficult to know whether these fungal isolates are truly endosymbionts of the intestinal tracts of the coleopteran larvae or are transitory inhabitants associated with host feeding habits. Hence, it is also possible that some of these microorganisms could be commensals, parasites, and facultative endosymbionts. They might even be using the insect as a dispersal mechanism [15, 53]. It is clear, however, that the overall taxonomic composition of the gut-inhabiting microbes and the proportion of lignocellulolytic-positive fungi seem to be particular to the larval microenvironment. The structure of this endosymbiotic community is distinguished from the fungal composition observed in other wood-related microhabitats such as the fungal populations in living plant tissues. They are also dominated by members of Ascomycetes, but they present a different abundance distribution of fungal families [54, 55]; decaying logs are dominated mainly by Basidiomycetes [56–58].

The analysis of the taxonomic composition of the bacterial isolates showed the presence of seven major phylogenetic classes, codominated by *γ*-Proteobacteria and Firmicutes. This finding is consistent with results obtained in similar studies [6, 13, 14, 44, 59]. Within the *γ*-Proteobacteria, the most abundant genera were* Enterobacter, Serratia, Acinetobacter,* and* Pseudomonas*. Interestingly,* Serratia* and* Pseudomonas* were isolated from all five coleopteran families studied;* Enterobacter* and* Acinetobacter* were present in four out of the five insect families, and they exhibited positive results in the lignocellulolytic assays, except for lignin degradation. Similar characteristics related to the degradation of lignocellulose and to fermentative metabolism were observed in* Bacillus,* the most abundant genus within Firmicutes [11, 60]. Together, these results support the notion that some species of fungi and bacteria, such as* Trichoderma*,* Serratia*,* Pseudomonas,* and* Bacillus,* can be common gut inhabitants of wood-feeding larvae in tropical forests, suggesting that certain affinities might have developed between the beetle host and its microbiota [41, 61–64].

When comparing the fungal and bacterial species composition among the beetle families, the plots of the Bray-Curtis distances and canonical correspondence analyses produced biologically meaningful clusters to group the environments that share similar microbial compositions. The first fungal cluster relates the microbiota associated with the guts of Cerambycidae, Passalidae, and Scarabaeidae. This is consistent with the observation of a high diversity of isolates from Cerambycidae that shared members of the fungal phyla Basidiomycota and Ascomycota with Passalidae and members of Zygomycota and Ascomycota with Scarabaeidae. The cluster formed by Tenebrionidae-Elateridae shared, in a lower proportion, members of the Basidiomycota and Ascomycota. The bacterial microbiota associated with Passalidae and Scarabaeidae also formed a cluster, sharing members of five major bacterial clades; microbiota of Cerambycidae, Elateridae, and Tenebrionidae shared members only of *γ*-Proteobacteria and Firmicutes.

The clustering analyses revealed that Cerambycidae presented a high diversity of fungi but not of bacteria, while Passalidae and Elateridae exhibited a high diversity of bacteria and moderate diversity of fungi. Scarabaeidae and Tenebrionidae contained a similar composition of both. These results suggest that the nature of the beetle host has an important effect on the phylogenetic diversity of its associated microbiota and that many factors can influence its configuration. These factors may include the biology of the host, the physical and chemical characteristics of the gut compartments, the feeding habits of the insects, and the microbial diversity associated with the environment in which the insect is living [23, 26, 65, 66].

Our results consistently showed that both the fungal and bacterial populations associated with the guts of beetle larvae are highly diverse in terms of the number of species obtained and in their phylogenetic composition. These microbial inhabitants could be forming complex consortia that would be acting synergistically to provide many of the nutritional needs of the beetle host. Some of these functions include the degradation and fermentation of lignocellulosic materials, as shown by the high percentage of fungal and bacterial genera that presented positive activities or by the production of proteins and other metabolites necessary for the development of the insect [25, 44, 67–69]. Furthermore, certain affinities for substrates can be expected according to the nature of the gut inhabitant. For example, members of the Basidiomycota could possibly degrade larger polymeric molecules, the Ascomycota deplete diverse lignocellulosic constituents, while the bacteria degrade and ferment the smaller monomeric and dimeric hexoses and pentoses produced by the fungal counterparts. The bacteria also likely use these sugars to produce other nutrients and metabolites. Consequently, the present work raises new lines of investigation concerning the existence of microbial consortia acting synergistically to provide the nutritional needs of the hosts, the nature of the ecological and evolutionary processes that contribute to ensure the fitness of the insect, and the mechanisms that rule the interactions among the fungi, the bacteria, and the beetle host.

## Supplementary Material

Statistical analysis: Canonical correspondence analysis of fungal and bacterial communities associated with the guts of five families of Coleoptera.

## Figures and Tables

**Table 1:** Description of the location and main environmental parameters of the 10 national parks of Costa Rica, where sampling was carried out. The selected environments are classified as tropical wet forest and cover most of the natural distribution of the five coleopteran families studied.

National park	Latitude Longitude	Altitude (m)	Mean temperature (°C)	Annual precipitation (mm)
Arenal	10°26′49′′ N 84°43′41′′ W	589	24	4000–5000

Barbilla	9°58′43′′ N 83°28′23′′ W	460	21	3000–4000

Braulio Carrillo	10°9′33′′ N 83°56′10′′ W	507	24	3500–4500

Carara	9°46′41′′ N 84°36′20′′ W	78	27	2500–3000

Hitoy Cerere	9°40′18′′ N 83°01′39′′ W	150	25	3000–4000

Piedras Blancas	8°41′56′′ N 83°12′29′′ W	198	28	5000–6000

Rincón de la Vieja	10°46′29′′ N 85°20′41′′ W	782	22	2500–3000

Tapanti	9°44′40′′ N 83°46′56′′ W	1287	19	6000–7000

Tenorio	10°42′25′′ N 84°59′22′′ W	727	22	3000–4000

Tortuguero	10°32′5′′ N 83°29′56′′ W	0	26	5000–6000

**Table 2:** Distribution of the number of larvae samples according to the insect family and national park. At each site, an approximate area of 150.000 m^2^ was explored for the presence of wood-feeding larvae. The number of insect groups found varied according to the natural condition of each forest. Each sample was composed of one to three individuals of the same species.

National park	Cer	Ela	Pas	Sca	Ten	Total
Arenal	1			3		4
Barbilla				6		6
Braulio	2	2		1		5
Carara	1		3	1		5
Hitoy Cerere			7			7
Piedras Blancas			1	2	1	4
Rincón de la Vieja	1		2			3
Tapanti	2		1	1	1	5
Tenorio		1		1	1	3
Tortuguero	1	2	2	1		6

Total	8	5	16	16	3	48

Cer: Cerambycidae, Ela: Elateridae, Pas: Passalidae, Sca: Scarabaeidae, and Ten: Tenebrionidae.

**Table 3:** Taxonomic distribution of the fungal isolates identified in this study. The number of isolates at the order and genera level is shown for each of the coleopteran families.

Order	Genus	Cer	Ela	Pas	Sca	Ten	Total
Botryosphaeriales	*Botryosphaeria*	1					1

Capnodiales	*Ramichloridium*				1		1

Chaetothyriales	*Cladophialophora*				2		2
	*Fonsecaea*	1					1
	*Rhynchostoma*			1			1

Diaporthales	*Phomopsis*					1	1

Eurotiales	*Aspergillus* ^*∗*^	1					1
	*Paecilomyces*	1		1	1		3
	*Penicillium*	3		1	1		5

Helotiales	*Scytalidium*		1				1

Hypocreales	*Acremonium*				1		1
	*Bionectria* ^*∗*^	1		1			2
	*Cladobotryum*				1		1
	*Cordyceps*				1		1
	*Cosmospora*					1	1
	*Elaphocordyceps*	1					1
	*Fusarium*	1			1		2
	*Gliocladiopsis*				1		1
	*Isaria*			1	1		2
	*Lanatonectria*		1				1
	*Mariannaea* ^*∗*^	1			2		3
	*Metacordyceps*			1			1
	*Metarhizium*	2		3	2		7
	*Nectria*			2			2
	*Neonectria*	1					1
	*Trichoderma* ^*∗*^	6	4	4	8	1	23

Microascales	*Graphium*	1	1				2
	*Pseudallescheria*				1	1	2
	*Scedosporium*				1		1

Ophiostomatales	*Sporothrix*		2		2		4

Pleosporales	*Leptosphaerulina*	1					1

Saccharomycetales	*Geotrichum* ^*∗*^			1	1		2

Xylariales	*Eutypa*			1			1
	*Pestalotiopsis*		1	1			2

Agaricales	*Coprinellus*		2			1	3

Polyporales	*Phlebia*					1	1
	*Trametes* ^*∗*^					1	1

Trichosporonales	*Trichosporon*	1		1			2

Mucorales	*Mucor* ^*∗*^	1			1		2
	*Rhizomucor*	1					1

Total		**25**	**12**	**19**	**29**	**7**	**92**

Cer: Cerambycidae, Ela: Elateridae, Pas: Passalidae, Sca: Scarabaeidae, and Ten: Tenebrionidae.

^*∗*^Genera that presented positive enzymatic activities in more than four pathways.

**Table 4:** Taxonomic distribution of the bacterial isolates obtained in this study. The number of isolates at the phylum and genera level is shown for each of the coleopteran families.

Class	Genus	Cer	Ela	Pas	Sca	Ten	Total
Actinobacteria	* Arthrobacter*			1			1
	* Cellulomonas*			1			1
	* Leifsonia*			1			1
	* Leucobacter*			3			3
	* Microbacterium*			1			1
	* Streptomyces*			2	5		7
	* Tsukamurella*		1				1

*α*-Proteobacteria	* Novosphingobium*			1			1
	* Rhizobium*			2			2

*β*-Proteobacteria	* Achromobacter*			1	1		2
	* Chromobacterium*		1				1

*γ*-Proteobacteria	* Acinetobacter* ^*∗*^		2	4	1	3	10
	* Alishewanella*	1					1
	* Azorhizophilus*				1		1
	* Citrobacter*				2		2
	* Dyella*			1			1
	* Enterobacter* ^*∗*^	4	4	1	3		12
	* Erwinia*	1					1
	* Klebsiella*	1			1		2
	* Kluyvera*		1				1
	* Pseudomonas*	2	1	1	4	1	9
	* Raoultella*				3	1	4
	* Salmonella*				2		2
	* Serratia*	4	2	2	2	2	12
	*Stenotrophomonas*			1	1		2

Firmicutes	* Bacillus* ^*∗*^	4	4	6	10	3	27
	* Enterococcus*			3	2		5
	* Lactococcus*	1		5	2		8
	* Lysinibacillus*		1	2	4		7
	* Paenibacillus*		1	1	1		3
	* Staphylococcus*		1		1		2

Flavobacteria	* Chryseobacterium*		1				1

Fusobacteria	* Sebaldella*			1			1

Total		**18**	**20**	**41**	**46**	**10**	**135**

Cer: Cerambycidae, Ela: Elateridae, Pas: Passalidae, Sca: Scarabaeidae, and Ten: Tenebrionidae.

^*∗*^Genera that presented positive enzymatic activities in more than four pathways.

**Table 5:** Results of the screening for lignocellulolytic activities. Fungal genera with positive results are shown in the upper group and bacterial genera in the lower group.

Genus	CMC	lignin	*β*-gluc	*β*-xyl	celob
*Aspergillus*	+		+	+	+
*Bionectria*	+	+	+	+	+
*Botryosphaeria*	+				
*Coprinellus*	+				
*Elaphocordyceps*	+				
*Eutypa*	+	+			
*Fusarium*		+			
*Geotrichum*	+		+	+	+
*Graphium*	+				
*Isaria*	+		+	+	
*Lanatonectria*	+				
*Mariannaea*	+	+	+		+
*Metacordyceps*	+		+	+	
*Mucor*	+		+	+	+
*Nectria*	+	+	+		
*Paecilomyces*	+	+			
*Penicillium*	+	+			
*Pestalotiopsis*	+				
*Phlebia*	+	+	+		
*Phomopsis*	+				
*Pseudallescheria*	+		+	+	
*Scytalidium*	+	+			
*Sporothrix*		+			
*Trametes*	+	+	+	+	+
*Trichoderma*	+	+	+	+	+
*Trichosporon*	+				

*Acinetobacter*	+		+	+	+
*Bacillus*	+		+	+	+
*Citrobacter*	+				
*Enterobacter*	+		+	+	+
*Enterococcus*	+		+		+
*Lactococcus*	+				
*Novosphingobium*	+				
*Paenibacillus*	+				
*Pseudomonas*	+				
*Rhizobium*	+				
*Serratia*	+				
*Stenotrophomonas*	+				
*Arthrobacter*	+				
*Microbacterium*	+				
*Streptomyces*	+				
*Tsukamurella*	+				

CMC: cellulase activity on carboxymethylcellulose, lignin: ligninolytic activity on Remazol Brilliant Blue R, *β*-gluc: *β*-glucosidase, *β*-xyl: *β*-xylanase, and celob: cellobiose hydrolase activity.
