# Supplementary figures and images for: Isolation of Fungi and Bacteria Associated with the Guts of Tropical Wood-Feeding Coleoptera and Determination of Their Lignocellulolytic Activities
Source: Int J Microbiol. 2015 Aug 26;2015:285018. doi: 10.1155/2015/285018 (PMC4563095; doi:10.1155/2015/285018)

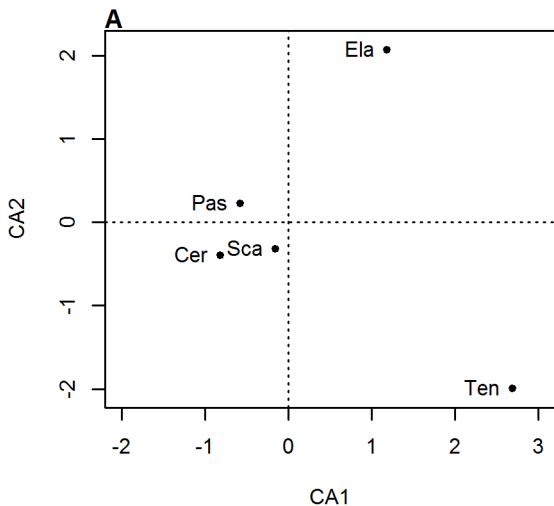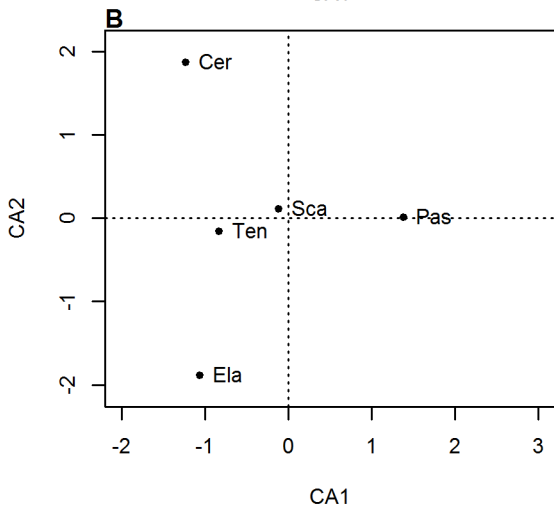

Supplement: Supplementary file 1 — Statistical analysis: Canonical correspondence analysis of fungal and bacterial communities associated with the guts of five families of Coleoptera. [file 285018.f1.pdf]
